# A Hidden Danger Behind the Self-Reported Intrauterine Pregnancy

**DOI:** 10.1016/j.acepjo.2025.100226

**Published:** 2025-07-15

**Authors:** Kenichiro Iga, Tomoyuki Shirahige, Shinji Yamada, Sunao Yamauchi

**Affiliations:** 1 Department of Emergency Medicine, Yuuai Medical Center, 50-5 Yone, Tomigusuku, Okinawa, 901-0224, Japan; 2 Departments of Emergency and Critical Care Medicine, Tokyo Bay Urayasu Ichikawa Medical Center, Urayasu, Chiba, Japan; 3 Department of Obstetrics and Gynecology, Yuuai Medical Center, 50-5 Yone, Tomigusuku, Okinawa, 901-0224, Japan

## 1 Patient Presentation

**Figure 1:**
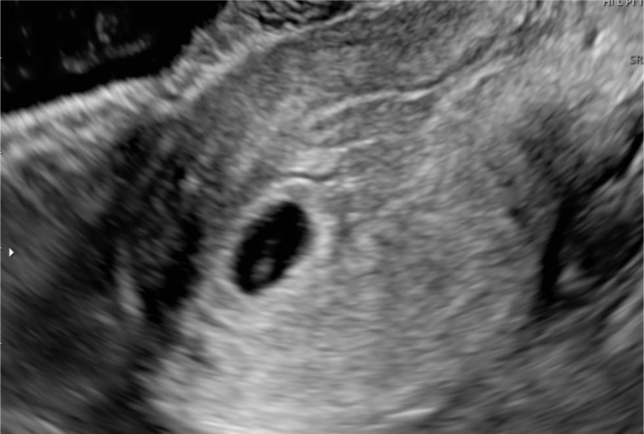
Transvaginal ultrasonography: a 12.1-mm gestational sac in the uterus.

**Figure 2:**
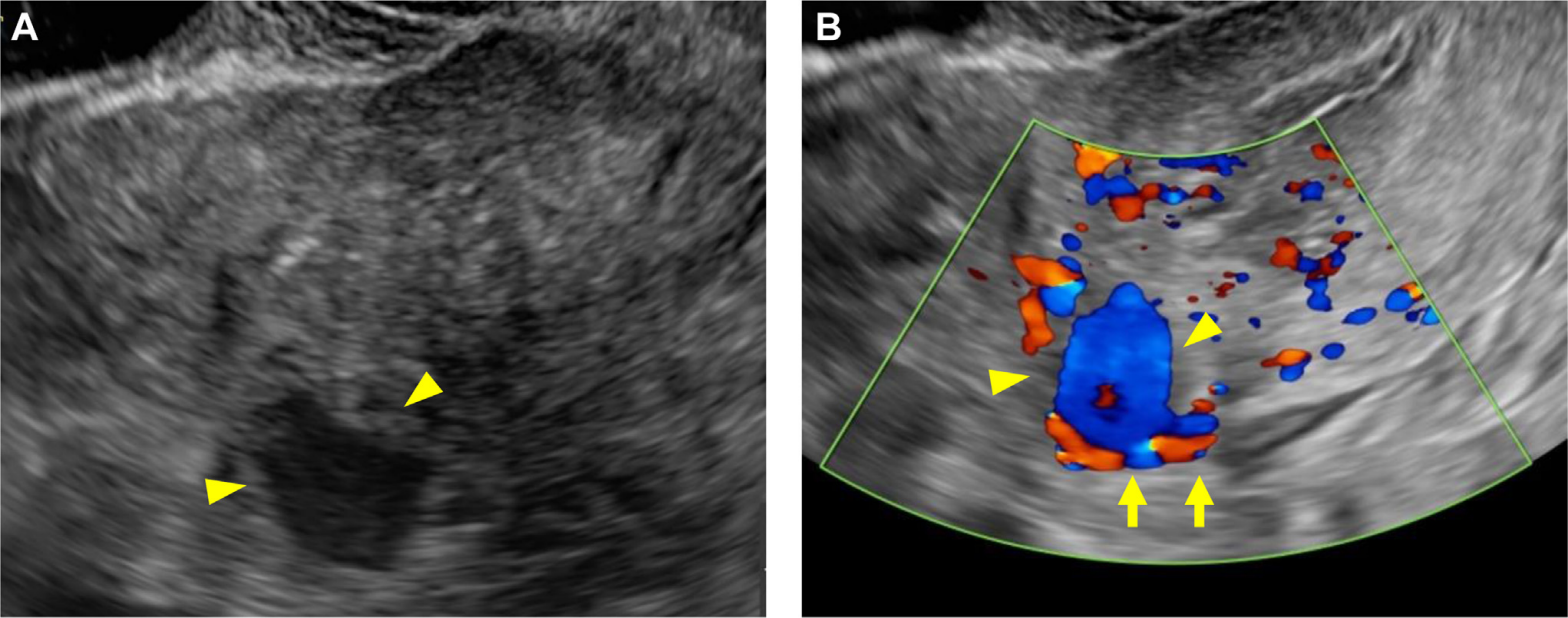
Transvaginal ultrasonography: (A) An 18.6-mm cystic mass (arrowheads) in the left uterine cornu; (B) A mass (arrowheads) with internal blood flow suggestive of hemorrhage (arrows) in the left uterine cornu.

A 32-year-old woman, gravida 3 para 0, at 5 weeks of gestation, presented to the emergency department with sudden onset of abdominal pain. She reported that she had an intrauterine pregnancy confirmed by her obstetrician. For this pregnancy, she had undergone in vitro fertilization and frozen embryo transfer with 2 embryos. She had a history of ectopic pregnancy and a left salpingectomy. On arrival, her vital signs were as follows: blood pressure, 89/61 mm Hg; heart rate, 102 beats per minute; and oxygen saturation, 100% without oxygen supplementation. Physical examination revealed pallor and right lower abdominal tenderness. Blood tests revealed a hemoglobin level of 8.3 g/dL. The serum β human chorionic gonadotropin level was 18,068.7 mIU/mL. Transabdominal ultrasonography showed significant free fluid extending from the liver surface to the pouch of Douglas. Transvaginal ultrasonography revealed a 12.1-mm gestational sac in the uterus and an 18.6-mm cystic mass in the left uterine cornu with internal blood flow suggestive of hemorrhage (Figs 1 and 2). She was considered to be in hypovolemic shock due to hemorrhage, and 4 units of red blood cells and 4 units of fresh frozen plasma were urgently transfused.

## 2 Diagnosis: Heterotopic Pregnancy

Heterotopic pregnancy has been reported to occur with a frequency of 1 in 30,000 pregnancies.^1^ However, in pregnancies resulting from assisted reproductive technology, the incidence is estimated to increase significantly, occurring in 0.7% to 1% of cases.^2^^,^^3^ Furthermore, in cases of multiple embryo transfer, the frequency increases in proportion to the number of embryos transferred.^4^ The presence of an intrauterine pregnancy does not exclude the possibility of a concurrent ectopic pregnancy, which can lead to misdiagnosis as a threatened miscarriage. One study found that approximately 6% of heterotopic pregnancies are missed during the initial ultrasound examination.^5^ A study reported that 15.4% of heterotopic pregnancies developed hypovolemic (hemorrhagic) shock.^6^ Thus, heterotopic pregnancy is a condition that can be missed and could be life-threatening, emphasizing the critical importance of timely diagnosis and intervention.

In our case, emergency laparoscopic surgery was performed and examination revealed swelling in the left uterine cornu, indicative of a possible interstitial pregnancy. Subsequently, a left cornual resection was performed. This case highlights the importance of considering heterotopic pregnancies in at-risk patients presenting with abdominal pain in early pregnancy, even when an intrauterine pregnancy is confirmed, as delayed diagnosis can lead to life-threatening complications such as hypovolemic (hemorrhagic) shock.

## Funding and Support

By *JACEP Open* policy, all authors are required to disclose any and all commercial, financial, and other relationships in any way related to the subject of this article as per ICMJE conflict of interest guidelines (see www.icmje.org). The authors have stated that no such relationships exist.
